# Childhood Risk Factors for Violent Ideations in Late Adolescence and Early Adulthood

**DOI:** 10.1002/cbm.2382

**Published:** 2025-03-22

**Authors:** Manuel Eisner, Denis Ribeaud, Laura Bechtiger, Aja Murray, Andrea Tam

**Affiliations:** ^1^ Institute of Criminology University of Cambridge Cambridge UK; ^2^ Jacobs Center for Productive Youth Development University of Zurich Zurich Switzerland; ^3^ Department of Psychology University of Edinburgh Edinburgh UK

**Keywords:** developmental criminology, mental health, sex differences, trait aggressiveness, Violent Ideations

## Abstract

**Background:**

Violent ideations (VIs) refer to thoughts, daydreams or fantasies of killing, inflicting serious physical harm or humiliating another person. Violent ideations are of particular interest at the intersection between mental health and violent behaviour. However, little is currently known about developmental trajectories of violent ideations in adolescence and early adulthood, and the extent to which childhood risk factors predict the likelihood of violent ideations.

**Aims:**

This study aims to address three key questions: (1) what are the developmental trends in violent thinking from ages 13 to 24, and how do they differ by sex? (2) To what extent can childhood risk factors predict VIs in late adolescence and early adulthood? (3) Are these associations sex‐specific?

**Methods:**

Data were collected from the z‐proso cohort study that is an on‐going population‐based longitudinal cohort study of 1555 participants. We use participant, teacher and parent reports to examine the extent to which childhood trait aggressiveness, poor impulse control, social rejection, an adverse family environment and violent media consumption predict the likelihood of violent ideations.

**Results:**

Descriptive analyses show that VIs strongly decline from late adolescence to early adulthood. We also find substantial between‐individual stability in VIs between ages 17, 20 and 24. Indicators of childhood aggressiveness, poor impulse control, social rejection, an adverse family environment and adult media consumption were found to consistently predict increased violent ideations among males. Among females, self‐reported aggressive behaviour, aversive parenting and a poor teacher–child bond had relatively strong associations with VIs. Overall, childhood risk factors were more predictive of VIs among male study participants than among females.

**Conclusions:**

The propensity to experience VIs declines between ages 15 and 24. The experience of VIs during late adolescence to early adulthood had long‐term associations with childhood risk factors indicative of general aggressiveness, low impulse control, social rejection, an adverse family context and violent media consumption. Most prospective associations were stronger for males than for females. This is consistent with the notion that a relatively stable violent potential is shaped in childhood for a larger proportion of males than females.

## 1 Background

Violent ideations (VIs) refer to thoughts, daydreams or fantasies of killing, inflicting serious physical harm or humiliating another person (Murray et al. 2017). VIs vary between individuals and over time in emotional intensity, amount of detail, imagined motivation, inflicted harm and frequency (Kenrick and Sheets 1993; Auvinen‐Lintunen et al. 2015; Murray et al. 2018). This can include scripts of unprovoked attacks but more typical scenarios entail a response to personal threat, humiliation and threats to a close friend or family member. VIs were therefore hypothesised to be part of an evolved psychological design that functions ‘to mobilise attention, rehearse scenarios, calculate consequences and motivate behaviour’ (Duntley and Buss 2011, 399).

VIs are of specific interest at the intersection between mental health and violent behaviour. In particular, VIs were found to be concurrently associated with a range of psychological difficulties and maladaptive behaviours. These include reactive and proactive aggression, hostility, anger‐related rumination, internalising symptoms, psychopathic traits, attention deficit hyperactivity disorder (ADHD) and low prosociality (Grisso et al. 2000; Murray et al. 2017). Additionally, violent and homicidal ideations that entail elements of retaliation and revenge are a possible consequence of victimisation experiences. For instance, peer victimisation and bullying victimisation were shown to predict increases in VIs during adolescence and early adulthood (Eisner et al. 2021; Spyropoulou and Giovazolias 2023; Wang et al. 2024). Furthermore, rehearsal of aggressive cognitive scripts was found to predict violent behaviour across various populations, including normative samples (Huesmann et al. 2017), psychiatric inpatients (Podubinski et al. 2017) and convicted offenders (Gilbert et al. 2013; Hosie et al. 2022). Among more extreme cases, persistent preoccupation with scripts of grievance, hatred, retaliation and homicide is frequently observed in convicted murderers (Burgason et al. 2024), terrorists (Clemmow et al. 2022), school shooters (Leary et al. 2003), and serial killers (Malmquist 1996). VIs are hence included in some clinically used risk assessment tools, such as the widely used HCR‐20^V3^ that includes an item on ‘thoughts, plans, desires, fantasies or urges to cause harm to others’ (Douglas et al. 2014).

Violent thought processes and scripts play a central role in several theoretical frameworks of crime and violence, including the general aggression model (Anderson and Bushman 2002), social learning theory (Akers 1998), the trait‐state model (Van Gelder and De Vries 2012) and the integrated cognitive antisocial potential (ICAP) theory (Farrington 2020). Within ICAP theory, violent scripts are seen as cognitive processes that proximally influence violent and delinquent decision‐making. These processes are shaped by an individual's long‐term antisocial potential which reflects the propensity to engage in antisocial behaviour. ICAP theory highlights childhood exposure to violent environments, personality traits such as impulsivity and socio‐economic strains as key factors contributing to the development of violent thinking patterns (Farrington 2020).

Despite the potential theoretical significance of violent ideations in developmental criminological theory, very few studies have empirically examined violent ideations from a long‐term developmental perspective. This study aims to address this gap using data from a large population‐based longitudinal cohort study. Specifically, we address three key questions: (1) what are the developmental trends in VIs from ages 15 to 24, and how do they differ by sex? (2) Do childhood risk factors (ages 7–11) predict VIs in late adolescence and early adulthood? (3) Are childhood predictors of VIs sex‐specific?

## 2 Childhood Risk Domains

We focus on five theoretically grounded childhood risk domains: trait aggressiveness, low impulse control, social rejection and poor social bonds, adverse family environment and problematic media usage.

### 2.1 Trait Aggressiveness

The tendency to have thoughts and fantasies about inflicting pain on others is sometimes seen as part of a wider syndrome of trait aggressiveness. It comprises interrelated behavioural, cognitive and emotional components, and has been consistently shown to have high homotypic continuity from early childhood to adulthood (Huesmann and Eron 1989; Oldehinkel and Ormel 2023; Obsuth et al. 2020). We therefore expect that behavioural, cognitive and attitudinal aspects of aggressiveness in children are predictive of VIs later in life (Murray et al. 2016).

### 2.2 Low Impulse Control

Low impulse control during childhood often manifests as poor mood regulation, irritability and disinhibition, and it is linked to anger and dysphoria in adolescence (Denson et al. 2012). We therefore investigate whether childhood symptoms of low inhibitory control, high sensation seeking and ADHD are associated with an increased risk of experiencing violent ideations at ages 17–24 (Schweizer et al. 2018).

### 2.3 Social Rejection

Social rejection and exclusion were shown to negatively influence well‐being in a variety of ways. This includes symptoms of psychological distress, poor physical health and negative thought patterns including anxiety, frustration, anger and rage (Twenge et al. 2001; Mulvey et al. 2017; Prinstein and La Greca 2004). We hence examine whether social rejection by peers and teachers in childhood is associated with VIs later in life.

### 2.4 Adverse Family Environment

Exposure to adverse family environments can promote aggression‐related cognitive and emotional processing in children (Widom 1989). For example, neglectful or abusive parenting can contribute to hypervigilance, hostile attribution biases, social learning of aggression and emotion dysregulation all of which may manifest in violent thought patterns (Lee and Hoaken 2007). We explore whether neglect, physical abuse and erratic parenting in childhood predict VIs in later life.

### 2.5 Problematic Media Usage

Finally, we investigate whether frequent media consumption and the consumption of adult media content in childhood predict the propensity for VIs in adolescence and adulthood. More specifically, chronic exposure to violent media content on TV, computer games and the internet during childhood has been argued to trigger long‐term processes of social learning that manifest in violent cognitions and desensitisation to violence (Anderson and Bushman 2018).

### 2.6 Sex‐Specific Risk Pathways

Throughout this study, we examine sex differences in the associations between childhood risk factors and adolescent or adult VIs. According to Moffitt's dual taxonomy of offending, males are more likely to follow a life‐course persistent trajectory because of greater exposure to childhood risk factors (Moffitt 1993; Odgers et al. 2008). Also, females may be buffered from the effects of childhood risks by their early acquisition of socio‐emotional and cognitive skills that offer protection against violent and antisocial tendencies (Bennett et al. 2005). We hence hypothesise that childhood risk factors are more strongly predictive of VIs among males than females.

## 3 The Study

The data for this study were collected from the Zurich Study on Social Development from Childhood to Adulthood or z‐proso. The study has been comprehensively described with respect to recruitment, key measurement domains, retention and sample characteristics in Ribeaud et al. (2022). It entails a cohort of 1675 first‐grade pupils who entered primary school in 2004 in one of 56 public schools in the City of Zurich. The main data collection waves were conducted at ages 7, 8, 9, 11, 13, 15, 17, 20 and 24 of the participants. Interviews with the primary caregiver were conducted when participants were ages 7, 8, 9 and 11. Teacher assessments were conducted annually between ages 7 and 12. Participants were interviewed in face‐to‐face interviews at ages 7, 8 and 9. From age 11 to 17, focal participants completed paper‐and‐pencil questionnaires in schools, and at ages 20 and 24 focal participants completed the questionnaire in a lab.

The current study uses data collected in the first four waves of z‐proso to construct the childhood predictors. Child participation in these waves was *n* = 1358 (age 7), *n* = 1333 (age 8), *n* = 1321 (age 9) and *n* = 1146 (age 11). Adolescent and early adult outcomes were constructed on the basis of waves 7–9 (ages 17–24). Participation in these waves was *n* = 1305 (age 17), *n* = 1180 (age 20) and *n* = 1159 (age 24). A total of 1555 participants (92.8% of the sample) contributed at least one wave of data. The sample is ethnically and culturally diverse. Over 65% of primary caregivers were born outside Switzerland representing over 70 countries.

## 4 Measures

Table A1 provides an overview of the measures used in this study, and their measurement characteristics.

Violent ideations were reported by study participants at ages 17, 20 and 24 using the violent ideations scale (Murray et al. 2017; Murray et al. 2018). The scale consists of 12 items relating to thoughts about killing another person (3 items), inflicting physical harm (7 items) or humiliating another person (2 items). The items describe situations with and without prior provocation, threat or attack. Respondents indicated the frequency of violent thoughts ‘in the past month’ on a five‐point Likert scale from ‘never’ to ‘very often’. Scale reliabilities were excellent (*α* > 0.90). For the descriptive analysis of developmental trends, we additionally report means of a 3‐item scale on reactive, instrumental and proactive aggressive thoughts administered at ages 13, 15 and 17 (Murray et al. 2016).

Fifteen variables were selected to represent five domains of childhood risk factors. Four variables represent behavioural, normative and cognitive aspects of child aggressiveness (teacher‐reported aggressive behaviour, child‐self‐reported aggression, moral neutralisation of violence and aggressive conflict coping); three variables relate to childhood impulsivity (low self‐control, high sensation seeking and ADHD symptoms); three variables relate to social rejection and poor social bonds (poor teacher–child bond, poor relationship with classmates and social isolation); two variables capture an adverse family environment (adverse parenting practices and poor parental involvement) and two variables represent measures of media usage (total time spent on media and adult media consumption). Finally, four variables represent the general socio‐demographic control variables (sex assigned at birth, socio‐economic status, migration background and neighbourhood disadvantage).

## 5 Method

We first conducted descriptive analyses to examine trends in levels of violent thinking patterns by sex at ages 13–24. For ages 13, 15 and 17 we rescaled the sex‐specific violent thoughts scale by regressing violent thoughts on the more comprehensive VI‐scale. For ages 17, 20 and 24 we calculated eta‐squared effect sizes in each wave to quantify the size of sex differences in VIs.

In a second step, we assessed the stability of violent ideations from late adolescence to early adulthood using autoregressive latent variable models over three time points (ages 17, 20 and 24) within a structural equation modelling framework. The validity of the measurement model for the 12‐item scale had been established in previous work (Murray et al. 2018). In each wave, VIs were modelled as a latent construct represented by the 12‐scale items. We included autoregressive paths where violent ideations at time *t* − 1 predicted violent ideations at time *t*. Missing data were handled using full information maximum likelihood (FIML).

In a third set of analyses, regression models were estimated to examine the relationships between each childhood characteristic measured and VIs at ages 17–24, adjusting for parental SES, family migration background and neighbourhood social disadvantage. Each assessed childhood characteristic was represented either by a manifest construct or as a latent variable represented by multiple constructs measured between ages 7 and 11. The models were estimated using the Lavaan package in R that facilitates the integration of latent variables in regression models (Rosseel 2012; R Core Team 2021). FIML was employed again to handle missing data (Little and Rubin 1989).

To examine whether the relationship between a childhood predictor and the propensity to experience violent ideations differed by sex, a multi‐group structural equation modelling (SEM) approach was applied. Two models were compared: in the constrained model, the regression paths from the childhood predictor to violent ideations were set to be equal across sex groups. In the unconstrained model, these paths were freely estimated for each group (i.e., separate coefficients for boys and girls). Model fit and differences were assessed using a chi‐squared difference test to determine whether constraining the regression paths led to a significant loss of fit.

We first estimated associations between each childhood risk factor and violent ideations separately for boys and girls, controlling for family socio‐economic status (SES), migration background and neighbourhood disadvantage (Models 1). Next, we re‐ran the models while additionally adjusting for children's general aggressive propensity at age 11 (Models 2). These models allowed us to estimate the unique contributions of childhood characteristics other than aggressive propensity on VIs during ages 17–24.

## 6 Results

**FIGURE 1:**
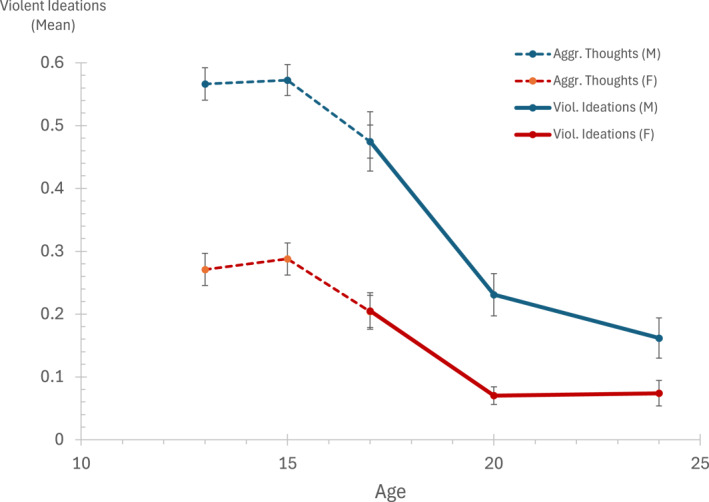
Developmental trends in violent thinking, past month, at ages 13–24, by sex. Scores at ages 17, 20 and 24 are means of the 12‐item violent ideations scale. Scores ate ages 13, 15 and 17 are based on a three‐item mean score of violent thoughts.

Mean levels of violent ideations at ages 17, 20 and 24 are graphically displayed in Figure 1.

For males and females violent ideations declined between ages 17 and 20. Between ages 20 and 24, the level of violent ideations declined further for males but remained unchanged for females. Furthermore, males had significantly higher levels than females of violent ideations at age 17 (adj. *η* ^2^ = 0.064, *p* < 0.0001), age 20 (adj. *η* ^2^ = 0.062, *p* < 0.0001) and age 24 (adj *η* ^2^ = 0.023, *p* < 0.0001). The corresponding Cohen's *d* effect sizes were *d* = 0.525, *d* = 0.506 and *d* = 0.268.

The autoregressive latent variable model for violent ideations over three time points (ages 17, 20 and 24) showed a reasonable fit (CFI = 0.860, TLI = 0.841, RMSEA = 0.07, SRMR = 0.06). Autoregressive coefficients indicated significant stability from age 17 to age 20 (*β* = 0.511, *p* < 0.001) and from age 20 to age 24 (*β* = 0.480, *p* < 0.001). The autoregressive coefficient between age 17 and age 24 was *β* = 0.387, *p* < 0.001.

The results in Table 1 show bivariate associations with childhood risk factors, adjusting for demographic variables.

**TABLE 1:** Childhood risk factors for violent ideations (Model 1): Associations by sex controlling for socio‐demographic background.

Childhood risk domain	Violent ideations, age 17–24				*χ* ^2^ M‐F difference
	Male		Female		
	B (SE)^a^	Beta	B (SE)^a^	Beta	
Aggressive propensity					
1. Aggressive behaviour, ages 7–11, TR	0.226 (0.038)	0.340***	0.001 (0.023)	0.003	26.27 (*p* < 0.0001)
2. Aggressive behaviour, age 11, SR	0.346 (0.054)	0.386***	0.129 (0.030)	0.292**	12.16 (*p* = 0.0005)
3. Aggressive conflict coping, age 11, SR	0.249 (0.040)	0.359***	0.031 (0.022)	0.087	21.88 (*p* < 0.0001)
4. Moral neutralisation of violence, age 11, SR	0.304 (0.048)	0.374***	0.046 (0.022)	0.127*	23.20 (*p* < 0.0001)
Overall aggressive propensity (latent score of 1–4)	1.044 (0.197)	0.487***	0.493 (0.202)	0.267*	2.25 (*p* = 0.1188)
Poor impulse control					
5. Low self‐control, age 11, SR	0.332 (0.049)	0.391***	0.013 (0.020)	0.039	35.92 (*p* < 0.0001)
6. Sensation seeking, age 7, SR	0.370 (0.093)	0.200***	0.042 (0.034)	0.066	10.96 (*p* = 0.0009)
7. ADHD symptoms, age 7–11, TR	0.121 (0.027)	0.241***	0.009 (0.012)	0.044	14.52 (*p* = 0.0001)
Social rejection and poor social bonds					
8. Poor teacher bond, age 11, SR	0.223 (0.038)	0.335***	0.047 (0.015)	0.179***	18.53 (*p* < 0.0001)
9. Poor relationship with peers, age 11, SR	0.120 (0.038)	0.179**	0.022 (0.014)	0.087	5.89 (*p* = 0.0153)
10. Isolated, age 7–11, TR	0.150 (0.037)	0.238***	0.014 (0.013)	0.068	12.47 (*p* = 0.0004)
Negative parenting					
12. Aversive parenting, age 11, SR	0.221 (0.058)	0.224***	0.095 (0.024)	0.267***	4.03 (*p* = 0.0445)
13. Low parental involvement, age 11, SR	0.216 (0.052)	0.244***	0.025 (0.025)	0.074	11.74 (*p* = 0.0006)
Media consumption					
14. Adult media consumption, age 11, SR	0.090 (90.021)	0.257***	0.005 (0.009)	0.030	13.54 (*p* = 0.0002)
15. Total time media use, age 11, SR	0.174 (0.035)	0.299***	0.015 (0.014)	0.067	17.47 (*p* < 0.0001)

Abbreviations: PR = parent report; SR = child self‐report and TR = teacher report.

^a^ Controlling for family SES, migration background and neighbourhood disadvantage.

* *p* < 0.05.

** *p* < 0.01.

*** *p* < 0.001.

In the domain of childhood trait aggressiveness, higher teacher‐assessed aggressive behaviour, more self‐reported aggressive behaviour, higher moral neutralisation of violence and aggressive conflict coping were all strongly associated with an increased tendency to experience violent ideations at ages 17–24 among males. Among females, only self‐reported aggressive behaviour was significantly associated with later violent ideations. In the domain of childhood impulsivity, low self‐reported self‐control, high sensation seeking and higher teacher‐reported ADHD symptoms were predictive of more violent ideations among males. Among females, only childhood sensation‐seeking predicted VIS tendencies. In the risk domain of social rejection, a poor social bond with the teacher, poor relationship with peers in class and being socially isolated were correlated with a higher tendency to experience violent ideations among males. For females, a poor social bond with the teacher was predictive of more violent ideations. In the domain of parenting and family context, a negative parent‐reported family climate, self‐reported aversive parenting and low parental involvement were predictive of higher violent ideations at ages 17–24 among males. Among females, experiences of aversive parenting were associated with a higher tendency to experience violent ideations. Finally, in the domain of media consumption, both the consumption of adult (restricted to > 18 years) media content and the total time of media usage predicted male violent ideations but not female violent ideations. Twelve of the 15 childhood risk factors were more strongly associated with violent ideations among males than among females.

Table 2 shows childhood risk factors predictive of violent ideations after accounting for overall childhood aggressiveness.

**TABLE 2:** Childhood risk factors for violent ideations (Model 2): Associations by sex controlling for childhood aggressive propensity and socio‐demographic background.

Childhood risk domain	Violent ideations, ages 17–24				*χ* ^2^ M‐F difference
	Male		Female		
	B (SE)^a^	Beta	B (SE)	Beta^a^	
Poor impulse control					
Low self‐control, age 11, SR	0.117 (0.067)	0.137	0.055 (0.025)	0.155*	6.25 (*p* = 0.012)
Sensation seeking, age 7, SR	0.312 (0.093)	0.158***	0.053 (0.035)	0.080	6.76 (*p* = 0.0093)
ADHD symptoms, age 7–11, TR	0.03 (0.030)	0.068	−0.00 (0.013)	−0.025	1.62 (*p* = 0.2030)
Social rejection and poor social bonds					
Poor teacher bond, age 11, SR	0.114 (0.041)	0.165**	0.033 (0.017)	0.119*	3.36 (*p* = 0.0667)
Poor relationship with peers, age 11, SR	0.034 (0.038)	0.048	−0.008 (0.015)	−0.032	0.39 (*p* = 0.5352)
Isolated, age 7–11, TR	0.027 (0.009)	0.149**	0.010 (0.014)	0.044	5.29 (*p* = 0.0215)
Negative parenting					
Aversive parenting, age 11, SR	0.067 (0.060)	0.064	0.079 (0.025)	0.218**	0.03 (*p* = 0.8543)
Low parental involvement, age 11, SR	0.179 (0.050)	0.192***	0.014 (0.020)	0.040	9.24 (*p* = 0.0024)
Media consumption					
Violent media consumption, age 11, SR	0.034 (0.022)	0.093	−0.001 (0.010)	−0.008	2.18 (*p* = 0.1395)
Total time media use, age 11, SR	0.070 (0.038)	0.115	0.003 (0.014)	0.012	2.75 (*p* = 0.0974)

Abbreviations: PR = parent report; SR = child self‐report and TR = teacher report.

^a^ Controlling for overall aggressive propensity, family SES, migration background and neighbourhood disadvantage.

* *p* < 0.05.

** *p* < 0.01.

*** *p* < 0.001.

Among male participants, sensation seeking, poor teacher bond, being isolated and low parental involvement were unique predictors of VIs. Among female participants, unique prospective predictors were low self‐control, low teacher bond and experiences of aversive parenting.

## 7 Discussion

In this study, we examined the trajectories and childhood predictors of VIs during late adolescence and early adulthood in a normative longitudinal cohort sample.

Results on developmental trajectories showed that VIs are highest in mid adolescence, and decline steeply during late adolescence and the transition to early adulthood. The late adolescence decline in VIs aligns with trends of related indicators in this sample. This includes declining trajectories of serious assault (Steinhoff et al. 2023), violent extremist attitudes (Nivette et al. 2021), legal cynicism (Nivette et al. 2020), self‐regulation and general delinquency (Murray et al. 2021). It is also in line with a peak in self‐reported physical aggression around age 15 found in other samples (e.g., Brame et al. 2001). Furthermore, in an earlier study, Guerra et al. (2003) established an increase in aggressive fantasies between ages 7 and 10, and a peak at ages 10–12. Furthermore, a study of homicidal ideation diagnoses in a US‐based nationwide emergency department sample showed an age‐homicidal ideation curve with an increase from age 5 to age 14/15, and a subsequent decrease until age 17 (Vaughn et al. 2020). Taken together, emerging evidence suggests a normative age curve of violent ideations. It is characterised by an increase in late childhood and early adolescence, a peak in mid‐adolescence and a decline until early adulthood.

We also found sex differences in the likelihood of thoughts about killing, beating or humiliating another person. Notably, the effect size of sex declined from a medium effect size of around Cohen's *d* = 0.52 in late adolescence to a small effect size of Cohen's *d* = 0.27 in early adulthood mainly reflecting a decline in male VIs (Murray et al. 2017). Sex differences in homicidal and more generally violent ideations were found in most but not all studies of adolescent and early adult samples (Auvinen‐Lintunen et al. 2015; Vaughn et al. 2020; Guerra et al. 2003). For example, in a Chinese sample of 13.5 year olds, no sex differences in homicidal ideations were found (Li et al. 2023).

The findings also revealed substantial between‐individual stability in the tendency to experience VIs. The rank‐order stability coefficients were, for the entire sample, 0.52 (age 17–20) and 0.48 (age 20–24). These estimates are similar to those found in other studies of violent and hostile thinking patterns between adolescence and early adulthood (e.g., Caprara et al. 2007). They may be interpreted as evidence that VIs comprise a trait‐like component in the sense of a fairly stable pattern of violent thoughts and feelings that may be part of an overarching tendency to behave aggressively across situations and over time (Bushman 1995; Huesmann et al. 1984). However, it is important to bear in mind that rank–order stability in cognitive and emotional tendencies may also result from continued exposure to environments such as an adverse family and school context or antisocial peer networks.

The examination of bivariate associations (adjusting for demographic and socio‐economic variables) suggested significant associations between all five childhood risk domains and VIs. Among males, childhood low self‐control, self‐reported aggressive behaviour, moral neutralisation of violence, teacher‐rated aggressive behaviour and aggressive conflict coping had the strongest associations with VIs in late adolescence and early adulthood. Among females, self‐reported aggressive behaviour, aversive parenting, and a poor teacher–child bond had relatively strong associations with VIs. Importantly, we found that childhood risk factors are more strongly predictive of VIs among male study participants than among females. More specifically, 12 out of 15 risk factors showed significant sex differences in the strength of the prospective associations.

Two key explanations may account for these striking sex differences in predictive patterns. Following dual taxonomy theory by Moffitt (1993), the stronger prospective correlations for males may be interpreted as evidence of the over‐representation of male children in the life‐course persistent trajectory (Moffitt and Caspi 2001). This trajectory group is believed to have neuropsychological vulnerabilities that continue into adulthood and are highly resistant to social influences. In particular, boys are more likely to experience childhood neurobiological deficits associated with the processing of impulses (hyperactivity, impulsivity or poor emotion regulation). Chronic violent thought patterns during adolescence may be particularly likely among this subgroup. In contrast, theories of gender‐specific socialisation and social learning provide a different possible explanatory framework (e.g., Bennett et al. 2005). In this perspective, males at primary and secondary school age are far more likely to be exposed to, and identify with, violent male role models and pro‐violence beliefs. Hence, the stronger prospective correlations of childhood risk factors may be indicative of stronger cascading dynamics among male adolescents that link childhood risks to masculine thinking patterns about revenge, fighting strength, toughness and dominance.

In a final step, we examined prospective associations between childhood risks and VIs after controlling for the underlying aggressive propensity that may be interpreted as indicating homotypic continuity. Overall, prospective associations for all remaining risk factors are attenuated and sex differences in effect sizes were smaller. For males, we found that sensation seeking, a poor teacher–child bond, being socially isolated and low parental involvement had unique associations with VIs. Three of these variables may be interpreted as indicating experiences of social rejection by teachers, peers and parents. They may suggest that males are more likely than females to follow a developmental pathway that links early experiences of interpersonal rejection to hostile cognitions, anger and other‐directed aggression which may manifest as thoughts about homicide, physical harm and humiliation later in life (Leary et al. 2006). For females, we found that aversive parenting, low self‐control, and a poor teacher–child bond were uniquely associated with later IVs. Three of the indicators of social rejection by peers, teachers and parents contribute to the prediction of violent ideations later in life. The findings bear on developmental theories such as Farrington's (2020) ICAP theory which recognises the importance of developmental processes that lead to cognitive scripts linked to violence. In line with expectations formulated within ICAP theory, we found that violent ideations in adolescence and early adulthood are predicted by childhood domains that reflect a long‐term antisocial potential, poor bonds with adults, and a harsh family environment. Additionally, with the ICAP theory, we found a long‐term decline in violent ideations after mid‐adolescence (Farrington and McGee 2017). However, the striking sex differences in the strength of prospective risk factors were not expected, and require further investigation.

## 8 Conclusions

The propensity to experience VIs tends to decline between ages 15 and 24. At all ages, it is significantly higher among males than females, and shows a considerable level of rank‐order stability over time. The experience of VIs during late adolescence to early adulthood had long‐term associations with childhood risk factors indicative of general aggressiveness, low impulse control, social rejection, an adverse family context and violent media consumption. Most prospective associations were stronger for males than for females. This is consistent with the notion that a relatively stable violent potential is shaped for a larger proportion of males than females.

## Ethics Statement

Given the minimally intrusive nature of the study design, questions and intervention, ethical approval was not initially required in accordance with Swiss regulations. Since 2017, ethics approval for the main study has been provided by the Ethics Committee at the Faculty of Arts and Social Sciences of the University of Zurich.

## Conflicts of Interest

The authors declare no conflicts of interest.

## Data Availability

The data that support the findings of this study are available on request from the corresponding author. The data are not publicly available because of privacy or ethical restrictions.

## Appendix A

**TABLE A1:** Overview of constructs.^a^

Construct	Measure description	Cronbach's *α*	Scale/coding
Violent ideations			
Violent ideations	Violent ideations were assessed with 12 items from the violent ideations scale (at ages 17, 20 and 24)	0.91 (age 17)	Mean score five‐point scale from ‘never’ to ‘very often’
		0.90 (age 20)	
		0.93 (age 24)	
Aggressive thoughts	Aggressive thoughts were assessed at ages 13, 15 and 17 with 3 items on aggressive thoughts in the past month relating to reactive violence, instrumental violence and proactive verbal aggression	0.57 (age 13)	Mean score four‐point scale from ‘never’ to ‘almost every day’
		0.50 (age 15)	
		0.55 (age 17)	
Demographics			
Child sex	Child sex assigned at birth was available from the official school records	N/A	Binary variable (0 = female; 1 = male)
Family SES	Family SES was assessed using the international socio‐economic index of occupational status (ISEI; Ganzeboom et al. 1992) that assigns a score to occupations based on their socio‐economic characteristics and required educational level. A composite score was created based on information from when participants were 11, 13 and 15 to maximise information	N/A	16 (e.g., unskilled worker) to 90 (e.g., judge)
Migration background	Migration background was assessed using parental country of birth reported by focal participants. A composite score was created based on information from when participants were 11, 13 and 15 to maximise information	N/A	Binary variable (0 = at least one parent born in Switzerland; 1 = both parents born abroad)
Neighbourhood disadvantage	Neighbourhood disadvantage was measured on the basis of 2000 census data at the level of output areas as a factor score with ‘% unemployed’, % unqualified occupations, % primary education as highest education level, low % ‘management roles’ as variables with highest factor loadings	N/A	Standardised factor score
Aggressive propensity			
Aggressive behaviour, ages 7–11, TR	Aggressive behaviour was assessed with 11 items from the social behaviour questionnaire at ages 7, 8, 9, 10 and 11 (Murray et al. 2017)	0.93	A latent score across five waves was computed. Item responses in each wave were on a five‐point scale from ‘never’ to ‘very often’
Aggressive behaviour, age 11, SR	Aggressive behaviour was assessed with 11 items from the social behaviour questionnaire at age 11	0.87	Item responses were a five‐point scale from ‘never’ to ‘very often’
Aggressive conflict coping, age 11, SR	Aggressive conflict coping was measured with 4 items rated by focal participants. Participants were asked how often, in case of a dispute with others, they engaged in activities such as threatening the other person with punches	0.69	Mean score of five point Likert scale five‐point scale from ‘never’ to ‘very often’
Moral neutralisation of violence, SR	Moral neutralisation was measured with a 12‐item scale that measures how the respondents justify violent behaviour. Items include, for example, ‘Many problems can be solved with violence’, it is alright to fight to protect your friends	0.87	Four‐point scale from ‘very untrue’ to ‘very true’
Poor impulse control			
Low self‐control, age 11, SR	10 items from the Grasmick et al. (1993) Self‐Control Scale were administered to measure self‐control. Higher values represent lower levels of self‐control	0.75	Mean score: Four‐point Likert scale from ‘fully untrue’ to ‘fully true’
Sensation seeking, age 7, SR	Sensation seeking at age 7 was measured with a board game where children chose a high risk and a low risk option	N/A	Sum score of ‘high risk’ choices across 9 situations
ADHD symptoms, TR, age 7–11	ADHD symptoms were teacher‐reported on 9 items from the social behaviour questionnaire where teacher rated their child's inattentive and hyperactive behaviours (Murray et al. 2017)		A latent score across five waves was computed. Item responses in each wave were on a five‐point scale from ‘never’ to ‘very often’
Social role in classroom			
Age 11, poor teacher bond, SR	Teacher–child bond was measured with 3 items on whether the teacher is fair, the teacher supports the child, and whether the child gets along with the teacher	0.78	Four‐point scale from ‘very untrue’ to ‘very true’
Age 11, poor relationship with peers, SR	Class bonding was assessed with 3 items on whether the child gets along with classmates, whether classmates are nice with the child and whether class community is good	0.77	Four‐point scale from ‘very untrue’ to ‘very true’
Age 7–11, isolated, TR	In each teacher assessment at ages 7, 8, 9, 10 and 11, the teacher was asked to assess the extent to which the child is isolated among its peers, single item	N/A	Mean score: 5‐point scale from ‘never’ to ‘very often’
Negative parenting			
Aversive parenting, age 11, SR	Participants reported exposure to aversive parenting on 6 items from the Alabama parenting questionnaire (APQ; including corporal punishment, verbal abuse and harsh/inconsistent parenting)	0.63	Mean score: 4‐point scale from ‘never’ to ‘always’
Low parental involvement, age 11, SR	Low parental involvement was child rated on 6 items (e.g., ‘Your parents show interest in what you do’; when you have problems, you can go to your parents) of the APQ. The scale was inverted	0.65	Mean score: 4‐point scale from ‘never’ to ‘often’
Media use			
Media use total time, age 11, SR	Mean time spent on media use was assessed with six questions on time spent on Internet, watching TV/DVD and playing computer games during weekdays and on Saturday	0.80	Five point scale from ‘never’ to ‘over 3 h’. The average almost of the six items was computed
Violent media use, age 11, SR	Violent media use was assessed with three items on having ever watched adult rated (18+) ‘horror films’, ‘action films’ or played adult rated computer games	N/A	The sum of ‘yes’ responses was computed, range 0–3

^a^ See z‐proso Project Team (2024) for a full documentation of instruments and procedures.

Abbreviations: PR = parent report; SR = self‐report; TR = teacher‐report.
